# Future of bNAbs in HIV Treatment

**DOI:** 10.1007/s11904-025-00744-1

**Published:** 2025-05-27

**Authors:** Pablo Tebas

**Affiliations:** Division of Infectious Diseases, Hospital of the University of Pennsylvania, Philadelphia, PA USA

**Keywords:** HIV, Broadly neutralizing antibodies, Antiretroviral therapy, HIV prevention, Viral resistance

## Abstract

**Purpose of Review:**

Broadly neutralizing antibodies (bNAbs) represent a novel approach to HIV treatment, prevention, and cure strategies. As research advances, the clinical application of bNAbs continues to evolve. This review explores the potential role of bNAbs in HIV management, addressing their mechanisms of action, current limitations, and future directions.

**Recent Findings:**

Recent studies have demonstrated that bNAbs can effectively neutralize a broad range of HIV strains by targeting conserved epitopes on the viral envelope. Clinical trials have shown that bNAb combinations can maintain viral supression in the absence of antiretroviral therapy (ART), though pre-existing resistance remains a major challenge. Strategies such as Fc engineering and alternative delivery mechanisms (e.g., AAV, mRNA, DNA) are being explored to enhance bNAb efficacy and durability. Despite promising data, bNAbs have not yet demonstrated superior effectiveness compared to existing ART or pre-exposure prophylaxis (PrEP) options.

**Summary:**

While bNAbs offer exciting possibilities for long-acting HIV therapy, their widespread use is limited by logistical challenges, high production costs, and pre-existing viral resistance. The future of bNAbs may lie in combination strategies with small-molecule antiretrovirals in maintenance strategies, genetic delivery systems, and vaccine-based approaches to induce endogenous bNAb production. Further research is needed to refine these strategies and determine the optimal role of bNAbs in HIV care.

## Introduction

HIV infection remains a critical global health issue, with nearly 40 million people affected worldwide as of the end of 2023 [1]. While antiretroviral therapy (ART) has significantly lowered HIV-related morbidity and mortality there is still a need for new treatment options. Current advancements in ART are moving toward long-acting formulations with extended half-lives, such as injectable cabotegravir and rilpivirine [2, or lenacapavir [3, allowing for less frequent dosing and improved adherence. Broadly neutralizing antibodies (bNAbs) have emerged as a promising tool in the mangement of HIV infection [4]. These “biological natural products” mimic the body’s immune response by targeting the HIV envelope and neutralizing a wide range of viral HIV strains. HIV bNAbs are being explored not only for their potential in long-acting HIV therapy but also for preventive applications like PrEP, prevention of maternal-child transmission, and cure-related studies aiming to eliminate or reduce the viral reservoir.

## What Is a Broadly Neutralizing Antibody (bNAb)?

Neutralization of a virus refers to the ability of an antibody to prevent the virus from infecting the host cell. This process is usually measured in vitro, where neutralizing antibodies (nAbs) bind to specific proteins on the surface of the virus, blocking the virus’s ability to attach to the cells in tissue culture and hopefully the host cells in vivo [5, 6, 7]. In contrast, not all antibodies that bind to virus have neutralizing capabilities. Some antibodies may attach to the virus but do not prevent it from entering and infecting cells. These non-neutralizing antibodies may still play a role in the immune response by marking the virus for destruction by other immune cells (a process known as opsonization) or triggering other immune mechanisms like antibody-dependent cellular cytotoxicity (ADCC) when the proteins are expressed in the cell membrane.

In the case of HIV, bNAbs target the viral envelope (Env) protein, which forms spike-like structures on the virus’s surface. By binding to these spikes, bNAbs block or interferes with the virus from attaching to the CD4 receptors on human immune cells, ultimately preventing infection [5].

bNAbs are unique in their ability to neutralize a broad range of HIV strains by binding to highly conserved regions of the virus that are less prone to mutation. Currently, five primary regions on the HIV Env trimer are targeted by available bNAbs including the CD4 binding site, trimer apex (V1/V2 loops), high-mannose patch, gp120-gp41 interface (including the fusion peptide) and the membrane proximal region (MPER) [8].

## Engineering bNAbs for Desired Characteristics

Antibody engineering encompasses a range of strategies aimed at enhancing the therapeutic potential of monoclonal antibodies, especially through modifications to specific antibody regions. These modifications can increase antibody binding affinity, extend half-life, and improve immune system interactions or effector functions like antibody-dependent cellular cytotoxicity (ADCC) and complement-dependent cytotoxicity (CDC) [9].

Various Fc region modifications alter mAb effector functions. For example, the L234A and L235A mutations (the LALA modification) in the anti-SARS-CoV-2 antibody etesevimab disable FcγRI and FcγRII binding to reduce the risk of antibody-dependent enhancement (ADE) [10].

Engineering mAbs to extend their half-life involves targeting interactions with the neonatal Fc receptor (FcRn), responsible for recycling IgG antibodies back into the bloodstream. This recycling process primarily occurs within endothelial and epithelial cells throughout the body. FcRn binds antibodies at acidic pH (e.g., pH 6.0) and releases them at physiological pH (7.4). Mutations such as M252Y/S254T/T256E (YTE) or M428L/N434S (LS) at the Fc–FcRn interface enhance binding to FcRn, increasing antibody half-life [11]. This approach has been applied in multiple HIV bNabs like VRC01LS, VRC01-523-LS, 3BNC117-LS, 10-1074-LS etc.

Antibody modifications also enhance therapeutic efficacy through glycoengineering, which adjusts Fc-linked glycan structures to optimize immune responses. Afucosylation, the removal of fucose residues from the Fc glycan, enhances ADCC by increasing binding to FcγRIIIa on immune cells like natural killer cells, a modification widely adopted in antibodies used in oncology indications [12].

Bispecific antibodies (bsAbs), engineered to target two different antigens or epitopes, are another innovative design enabling multi-targeted therapeutic effects. For example, bispecific T-cell engagers (BiTEs) connect T cells directly to cancer cells, boosting immune targeting in oncology [13]. Recent bsAbs developed for infectious diseases, such as a bsAb targeting the Niemann-Pick C1 (NPC1) protein and Ebola virus glycoprotein based on the idea of using a “Trojan horse” to deliver the antibody to the endosomal sites where the virus-receptor complex interact [14]. Advanced formats further include trispecific antibodies, like SAR441236, engineered for HIV treatment by targeting multiple sites on the HIV envelope to offer broader and more effective virus neutralization. The administration of the product in the A5377 study was safe and well tolerated, and has a favourable pharmacokinetic profile, however it had limited antiviral activity, most likely because the lack of potency of the individual specificities [15].

Beyond conventional mAbs, smaller antibody formats, such as nanobodies derived from camelids, offer advantages in antiviral applications. Camelids are a family of animals that includes species such as camels, llamas, alpacas, vicuñas, and guanacos. Camelids are notable because they naturally produce a unique type of antibody called heavy-chain-only antibodies. These single-domain antibodies, with extended binding regions, can bind to epitopes that are often inaccessible to conventional antibodies [16, 17].

## The Challenge of Pre-existing Resistance

In untreated PWH, bNAbs naturally develop through repeated cycles of viral escape and B cell responses [18]. Endogenous antibody responses can, on rare occasions, achieve durable virus control without antiretroviral therapy, but that is a very rare phenomenon [19]. As the virus continually evolves to escape neutralizing antibodies, the HIV Env protein rapidly mutates, creating a highly diverse viral population and a highly diverse immune reposnse against these envelopes [18]. This process is amplified in people with longer durations of HIV infection or poorly controlled viral replication, as the virus’s envelope protein (Env) becomes more diverse, driving the immune system to produce antibodies that, in turn, select for resistant viral strains. This diversity, which includes mutations in the CD4 binding site, trimer apex, high-mannose patch, gp120-gp41 interface, and membrane proximal region (MPER), poses a significant challenge for bNAb-based therapies.

We recently completed a study in the greater Philadelphia population and only 50% of chronically infected, virologically suppressed PWH were found to harbor virus sensitive to both bNabs 3BNC117 and 10-1074 [20]. While the criteria for determining sensitivity was somewhat arbitrary due to the limited data linking bnAb sensitivity testing to clinical outcomes, emerging clinical observations are providing insights into the frequency of this problem. For instance, the HVTN 704/HPTN 085 and HVTN 703/HPTN 081 studies, which involved over 5,000 participants, revealed that protection against infection by the CD4 binding antibody VRC01 was effective only against viruses sensitive to concentrations below 1 µg/mL [21]. In these trials, only 30% of infected participants in the placebo groups had viruses sensitive to VRC01, suggesting considerable pre-existing resistance in the communities studied, particularly in sub-Saharan Africa [21]. The constant viral mutation in HIV means that many PWH already harbor viral strains resistant to bNAbs, even before being exposed to these therapeutic products.

This issue of rapid viral evolution and preexistiing resistance is not unique to HIV. During the COVID-19 pandemic, several mAbs received emergency use authorization (EUA) for the early treatment of mild to moderate COVID-19, including bamlanivimab, etesevimab, casirivimab, and imdevimab were effective and decreased the risk of hopitalization and mortality [10]. However, at the population level, the virus quickly developed resistance to these antibodies, limiting their long-term effectiveness. While this resistance was concerning in COVID-19, it is even more pronounced in HIV, which has a much higher mutation rate than SARS-CoV-2.

## Current and Future Applications of bNAbs in HIV Management

- **Prevention**:

The use of bNAbs in prevention strategies holds promise, particularly given the success of passive immunization in treating and preventing various diseases including hepatitis A and B, rabies, measles, and other infections, particularly in immunocompromised people. More recently, during the COVID-19 pandemic, tixagevimab–cilgavimab (Evusheld) and more recently Pemivibart (Pemgarda) were utilized effectively to prevent infection in susceptible populations [22, 23].

The Antibody Mediated Protection (AMP) trials (HIV Vaccine Trials Network [HVTN] 704/HIV Prevention Trials Network [HPTN] 085 and HVTN 703/HPTN 081) evaluated the use of a bNab for the prevention of HIV [21]. These trials involved the infusion of VRC01, an HIV CD4 binding antibody, every 8 weeks over 20 months across two concurrent international studies, enrolling a total of 4,623 participants. One trial included cisgender men and transgender people in the United States, Switzerland, and South America, while the other focused on at-risk women in seven countries in sub-Saharan Africa.

The results were clear yet disappointing—neither trial demonstrated significant overall protection mediated by VRC01. However, there was some positive news: the intervention did prevent infection with antibody-sensitive HIV strains, achieving a protective efficacy of 75% against those viruses. The major limitation, however, was that only 30% of circulating viruses were sensitive to VRC01, meaning that pre-existing resistance in the community greatly restricted the efficacy of this approach [21].

As a result, it’s evident that an effective prevention strategy will require a combination of multiple antibodies. However, this approach presents further challenges due to the likely presence of circulating, pre-existing resistant viral mutants, which could limit the efficacy of even multi-antibody combinations. Moreover, the logistical challenges associated with intravenous infusions, stringent cold chain requirements, and the need to administer multiple antibodies with differing half-lives add complexity, casting uncertainty on the future viability of this approach in resource-limited settings. Even in regions with more resources, the logistical challenges of bNab administrations were highlighted during the COVID-19 pandemic, where the use of antibody-based therapies remained limited despite the possibility of intramuscular administration [24].

A significant additional hurdle for the future development of bNabs for this indication is the high effectiveness of alternative interventions, which are not only more affordable but also less logistically challenging. For example, oral tenofovir combined with XTC (FTC or 3TC) has been highly effective in preventing HIV transmission [25, 26, 27]. More recently, cabotegravir has been proven even more effective [28, 29, and a recent large study showed that lenacapavir administered once every six months was 100% effective in preventing HIV acquisition [30].

Given the near-perfect efficacy of these alternative interventions, future clinical trials for bNAbs for prevention would need to be designed as non-inferiority trials. This presents a substantial challenge, as a comparator with nearly 100% efficacy demands exceptionally large sample sizes to demonstrate comparable effectiveness. Consequently, the future of passive antibody administration for HIV prevention remains highly uncertain.

- **Treatment**:

BNabs have emerged as a promising therapeutic approach for HIV, because of their significant antiviral activity in viremic PWH [31, 32, 33, 34]. As single agents, and in combinations, several bNAbs have demonstrated the ability to reduce viral loads in PWH significantly [35]. When used as monotherapies, bNAbs, much like traditional antiretroviral drugs, lead to the rapid development of resistance in HIV because HIV’s high mutation rate enables the virus to quickly evolve and escape the selective pressure imposed by a single or multiple bNAbs [31, 32, 33, 34, 35].

Using bNAbs as monotherapy in people with suppressed HIV to maintain viral suppression has been unsuccessful [36]. Subsequently, combining two or more bNAbs in switch strategies from traditional ART has demonstrated the ability to control viral rebound in the absence of antiretroviral therapy. Notably, combinations like 3BNC117 and 10-1074 in adults and VRC01LS and 10-1074 in children have been shown to prolong viral suppression for months after discontinuing ART, highlighting the potential for bNAbs to maintain control over HIV without continuous drug intervention [37, 38, 39, 40]. However, the breakthrough rate of these strategies has been unacceptably high, even when combining 3 antibodies [40, revealing the vulnerability of these products, the problem of preexisting resistance, and the limitations of current sensitivity testing to predict who will be able to control viral rebound in the absence of traditional ART. The PhenoSense Monoclonal Antibody (mAb) Assay (Labcorp-Monogram Biosciences), conducted on PBMC or plasma samples, is the only CLIA-certified assay for evaluating sensitivity to monoclonal antibodies, but clinical validation remains limited [41].

An emerging strategy for long-acting injectable ART involves combining broadly neutralizing antibodies with small-molecule antiretroviral agents. In a recent phase 2 study, a combination of two intravenous long-acting bNAbs (zinlirvimab and teropavimab) with subcutaneously administered lenacapavir, a capsid inhibitor, showed promising results by maintaining viral suppression in 18 of 20 participants, although the follow-up was limited to 26 weeks [42]. More recently, a study combined VRC07-523LS with long-acting cabotegravir (CAB-LA), involving 74 adults with well-controlled HIV-1 for at least two years, all with a CD4 count ≥ 350 cells/mm³ and susceptibility to VRC07-523LS [43]. Participants first received an oral cabotegravir lead-in, followed by intravenous VRC07-523LS and monthly intramuscular CAB-LA for 48 weeks. Infusion reactions like chills, myalgia, and fatigue related to VRC07-523LS infusions occurred in 17% of the participants but were self-limited and did not lead to treatment discontinuation. Virologic suppression was maintained with a cumulative probability of virologic failure by week 44 of 7%, comparable to other maintenance strategies in switch studies [43]. These findings suggest that while long-acting, injectable ART combining one bNAb and one small-molecule antiretroviral can effectively suppress HIV-1, further progress will depend on gaining a deeper understanding of the factors that predict, drive, and result from viral breakthroughs.

Ongoing randomized studies, including EMBRACE (NCT05996471) and GS-5423/GS-2872 with lenacapavir (NCT05729568), will provide additional insights in this strategy. This is an area in which, almost certainly, long-acting bNAbs will be used in the future.

- **Cure Research**:

Despite growing insights into HIV reservoirs and latency, developing a cure—where people with HIV would no longer need daily ART—remains an elusive goal. BNAbs are being explored as key components of cure strategies due to their ability to target and eliminate infected cells through multiple mechanisms, including complement activation, antibody-dependent cellular phagocytosis (ADCP), and antibody-dependent cellular cytotoxicity (ADCC). Additionally, bNAbs may improve the host immune response through the “vaccinal effect, which occurs when bNAbs bind directly to viral antigens expressed on HIV-infected cells, leading to the formation of immune complexes. These immune complexes are recognized by Fc gamma receptors (FcγR) on antigen-presenting cells, such as dendritic cells, which enhances the uptake and presentation of viral antigens. This process may result in stronger and more robust HIV-specific CD8 + T-cell responses with enhanced cytotoxic activity [44].

Unfortunately, during successful antiretroviral treatment, there is minimal expression of HIV antigens on the surface of infected cells, making it difficult for the immune system to recognize and target these latent reservoirs. The results of using bNAbs to reduce the size of the HIV reservoir as single agents have been disappointing [45]. Latency-reversing agents are being investigated as a potential strategy to expose and eliminate the HIV reservoir by reactivating latent virus, making infected cells susceptible to immune system targeting, including passively delivered bNabs. Several classes of LRAs, including histone deacetylase inhibitors (HDACi), protein kinase C agonists, and toll-like receptor (TLR) agonists, have been tested in preclinical and clinical studies. Although some agents, such as HDAC inhibitors (e.g., vorinostat and romidepsin), have shown the ability to induce HIV transcription in latent cells, their impact on viral reservoir size and clearance has been minimal in clinical trials [46.Similarly, combination approaches using LRAs and bNAbs or immune-activating agents have shown modest results, with no significant reduction in the replication-competent reservoir [47, 48].

A randomized, open-label, phase 2 trial conducted at university hospitals in Denmark, Germany, and the USA evaluated whether combining 3BNC117 with romidepsin could delay viral rebound in PWH on long-term ART, compared to romidepsin alone [48]. Participants underwent two treatment cycles: romidepsin infusions at weeks 0, 1, and 2, followed by another cycle at weeks 8, 9, and 10. Those in the combination group received an additional 3BNC117 infusions before each cycle. ART was paused at week 24 to measure time to viral rebound. Of the 22 participants enrolled, 19 completed the study. Median time to viral rebound was 18 days in the combination group and 28 days in the romidepsin-only group—a statistically significant result (*p* = 0.0016), though not clinically meaningful.

Several ongoing trials are testing this approach: A5386 (NCT04340596) trial is a Phase 1 clinical study designed to evaluate the safety, tolerability, and efficacy of the IL-15 superagonist N-803, both alone and in combination with bNAbs 10-1074 and VRC07-523LS, to control HIV-1 during an analytic treatment interruption and has recently completed enrollment.

An alternative approach under consideration involves administering bNAbs to PWH while they are viremic, aiming to enhance the vaccinal effect and their effect on the HIV reservoir. The ECLAIR study, a phase 1b/2a open-label, multicenter randomized controlled trial, evaluated the impact of early intervention with the bNab 3BNC117 and romidepsin shortly after ART initiation [49]. 3BNC117, alone or in combination with romidepsin, accelerated plasma HIV-1 RNA decay and clearance of infected cells compared to ART alone, with notable enhancement of HIV-1 Gag-specific CD8 + T cell immunity in the combination group. During a 12-week analytical ART interruption, 3BNC117-treated PWH harboring sensitive viruses were significantly more likely to maintain virologic control. These findings suggest that early administration of bNAbs at ART initiation may enhance immune-mediated viral clearance and could represent a promising strategy to limit HIV-1 persistence.

A similar idea is being tested in the A5388 (NCT05719441) study. This is a double-blind, randomized, placebo-controlled clinical trial designed to evaluate whether the combination of VRC07-523LS and PGT121.414.LS, alongside ART, can induce HIV remission in people newly diagnosed with acute HIV infection. Participants are randomized to receive either ART with a bNAb or a placebo, with ART initiated immediately upon enrollment. The core hypothesis being tested is whether administering bNAbs during active HIV viremia—at the start of ART—can enhance the immune response and more efficiently reduce HIV reservoirs in people with smaller viral reservoirs, like those treated during acute HIV infection.

## Future Directions: Genetic Delivery of bNAbs and bNAb Inducing Vaccines

Delivering monoclonal antibodies as proteins poses significant logistical, manufacturing, and economic challenges. This approach requires a continuous cold chain for stability and intravenous administration, which restricts the feasibility of large-scale implementation across broad patient populations. To address these complexities, alternative delivery methods such as adeno-associated viruses (AAV), mRNA, and DNA-based systems are being explored. These approaches could, in theory, enable “one-and-done” solutions that provide sustained expression of these products over time, potentially eliminating the need for repeated administrations.

One notable example is a Phase 1, dose-escalation trial that evaluated the safety and tolerability of AAV8 delivery of the bNAb VRC07 in PWH [50]. In this study, a recombinant bicistronic AAV8 vector coding for both the light and heavy chains of VRC07 (AAV8-VRC07) was administered to eight participants who were on effective antiretroviral therapy with viral loads < 50 copies/mL. The primary endpoints were safety and tolerability, while secondary endpoints included the clinical effects on CD4 T cell count and viral load, and the persistence of VRC07 production. The study found that intramuscular injection of AAV8-VRC07 was safe and well tolerated, with measurable serum concentrations of VRC07 in all participants and stable levels in some people for up to three years. However, three out of eight participants developed a non-idiotypic anti-drug antibody (ADA) response directed against the Fab portion of VRC07. This ADA response appeared to reduce the production of serum VRC07, potentially impacting the antibody’s effectiveness in these participants. The results of a phase 1 study using mRNA to deliver monoclonal antibodies against Chikungunya virus was recently published [51]. DNA has been utilized in animal models for the delivery of HIV bNAbs [52]. DNA delivery of broadly neutralizing antibodies (bNAbs) involves injecting a DNA plasmid encoding the antibody genes directly into muscle tissue, typically followed by electroporation to enhance cellular uptake. Host cells then produce the bNAbs in vivo, providing sustained antibody expression without the need for repeated infusions of purified protein.

Showing dose-dependent expression following intravenous (IV) infusion at doses of 0.1, 0.3, or 0.6 mg/kg. The antibody exhibited a half-life of approximately 69 days, with sustained expression lasting up to 16 weeks at the higher dose levels. No anti-drug antibodies (ADA) were detected; however, transient increases in inflammatory markers—such as C-reactive protein, IL-6, complement, and IP-10—were observed shortly after dosing and resolved within 48 h. Participants in the 0.6 mg/kg group received corticosteroids to mitigate these inflammatory responses. Efforts are underway to develop vaccines that would induce new, broadly neutralizing HIV responses that could prevent viral infection. Several innovative vaccine designs target this goal by guiding the immune system toward producing bNAbs that can neutralize diverse strains of the virus. For instance, germline-targeting immunogens like the eOD-GT8 60mer aim to activate rare precursor B cells, with phase 1 trials showing successful induction of VRC01-class bNAb precursors in most recipients [53]. Antibody lineage-based designs further refine this approach by guiding B cell maturation along pathways that yield bNAbs [54]. Theoretically, a similar approach could be applied in PWH to enhance their autologous neutralizing activity to a level that would prevent viral rebound in the absence of ongoing therapy [55]. The A5322 study (NCT06680479) is testing this strategy.

## Conclusion

In conclusion, bNAbs hold significant potential to reshape HIV management across prevention, treatment, and cure-focused research. However, realizing this potential requires addressing substantial challenges, including pre-existing viral resistance, logistical complexities, and the demands of cold-chain storage and intravenous administration. Exploring alternative delivery mechanisms such as AAV, mRNA, DNA or vaccine inducing bNabs represents a crucial step forward in enhancing the practicality and accessibility of bNAb-based therapies. As these innovative approaches advance, bNAbs may offer transformative solutions in HIV care, bringing us closer to durable suppression and potentially curative strategies for HIV.

## Key References

1. **Burton DR.** Antiviral neutralizing antibodies: from in vitro to in vivo activity. *Nature Reviews Immunology* 2023; 23(11):720–734. DOI: 10.1038/s41577-023-00858-w.

Reviews the mechanisms of neutralizing antibodies in blocking viral infections and their translation to protective effects in vivo, emphasizing factors such as specificity, affinity, and effector functions in therapeutic development.

1. **Corey L**,** Gilbert PB**,** Juraska M**, et al. *Two Randomized Trials of Neutralizing Antibodies to Prevent HIV-1 Acquisition*. *NEJM* 2021; 384(11):1003–1014. DOI: 10.1056/NEJMoa2031738.

- Demonstrates that bNAbs provide protection against antibody-sensitive HIV strains but are ineffective against resistant variants.

2. **Mendoza P**,** Gruell H**,** Nogueira L**, et al. *Combination therapy with anti-HIV-1 antibodies maintains viral suppression. Nature* 2018; 561(7724):479–484. DOI: 10.1038/s41586-018-0531-2.

- Shows that dual bNAb therapy can sustain viral suppression in the absence of ART but with a high breakthrough rate.

3. **Julg B**,** Walker-Sperling VEK**,** Wagh K**, et al. *Safety and antiviral effect of a triple combination of HIV-1 broadly neutralizing antibodies: a phase 1/2a trial. Nature Medicine* 2024. DOI: 10.1038/s41591-024-03247-5.

- Highlights the limitations of bNAb-based therapy, with high resistance rates even when using triple bNAb combinations.

4. **Casazza JP**,** Cale EM**,** Narpala S**, et al. *Safety and tolerability of AAV8 delivery of a broadly neutralizing antibody in adults living with HIV: a phase 1*,* dose-escalation trial. Nature Medicine* 2022; 28(5):1022–1030. DOI: 10.1038/s41591-022-01762-x.

- Demonstrates the feasibility of using AAV-based genetic delivery of bNAbs, though challenges remain with immune responses.

## Data Availability

No datasets were generated or analysed during the current study.
